# Comparison of the Efficacy of Two-Week Vonoprazan Versus Lansoprazole-Based Quadruple Sequential Antibiotic Therapy in Eradicating Helicobacter pylori Infection: A Non-randomized Clinical Trial

**DOI:** 10.7759/cureus.52758

**Published:** 2024-01-22

**Authors:** Asma Nizam, Zao Iman Chaudary, Saad Ali Ahmad, Nimra Nawaz, Zainab Riaz, Aamir Shehzad, Muhammad Irfan Jamil

**Affiliations:** 1 Gastroenterology, Lahore General Hospital, Lahore, PAK; 2 Internal Medicine, Doctors Hospital, Lahore, PAK; 3 Internal Medicine, Services Hospital, Lahore, PAK; 4 General Medicine, Lahore General Hospital, Lahore, PAK; 5 Nephrology, Lahore General Hospital, Lahore, PAK

**Keywords:** infectious diseases, urea breath test, vonoprazan, proton pump inhibitor, helicobactor pylori

## Abstract

Aim: The prevalence of *Helicobacter pylori* is escalating in developing countries, exacerbated by unjustified antibiotic usage, which leads to increased resistance. This trend has been notably amplified since the COVID-19 pandemic. Consequently, the effectiveness of existing eradication regimens has been compromised. This study aimed to compare the efficacy of two weeks of vonoprazan-based quadruple sequential therapy and lansoprazole-based quadruple sequential therapy in treating *H. pylori* infection.

Methods: A non-randomized clinical trial was conducted over 18 months at the Department of Gastroenterology, Lahore General Hospital, Lahore, Pakistan. It included patients presenting with dyspepsia, as defined by the Rome IV criteria, and who tested positive on the urea breath test. Patients were divided into two groups, i.e., Group A and Group B. Group A patients received lansoprazole 30 mg + amoxicillin + tinidazole + tab. colloidal bismuth subcitrate for the first seven days, followed by lansoprazole + levofloxacin + azithromycin + colloidal bismuth subcitrate. Group B patients received vonoprazan + amoxicillin + tinidazole + colloidal bismuth subcitrate for the first seven days, followed by vonoprazan + levofloxacin + azithromycin + colloidal bismuth subcitrate. Both regimes continued for 14 days. Four weeks after 14 days of the treatment, an early morning urea breath test was conducted to evaluate the efficacy of the treatment. Patients were scheduled for follow-up visits at seven and 14 days post-treatment initiation to record adverse events and assess compliance with the treatment regimen. Patients who lost the follow-up and remained non-compliant to the medications were excluded from the final data analysis as per standard protocols of the per-protocol analysis.

Results: A total of 252 patients were included. In Group A and Group B, 6/126 (4.76%) and 8/126 (6.35%) of the patients were lost to follow-up, respectively. The non-compliance rate in Group A was 5/126 (3.97%), compared to Group B with 3/126 (2.38%). Finally, the per-protocol analysis of the results included 115 patients in each group. Baseline characteristics, including demographics, lifestyle, and clinical factors, were comparable between groups with p-values of 0.138 for age, 0.356 for gender, 0.126 for BMI, 0.495 for residence, 0.500 for water source, 0.866 for meal habit, 0.863 for smoking, 0.188 for nonsteroidal anti-inflammatory drug (NSAID) use, 0.145 for proton pump inhibitor (PPI) use, 0.213 for antibiotics, and 0.456 for treatment history. Both treatments effectively eradicated *H. pylori*, as determined by a negative urea breath test at four weeks post-treatment, with Group B showing a higher eradication rate of 96.5% compared to 92.2% in Group A, although the difference was not statistically significant (p = 0.153). There was no difference in adverse effects in both treatment groups (p-value > 0.05).

Conclusion: The study found that while the vonoprazan-based regime exhibited a slightly higher eradication rate of *H. pylori* compared to lansoprazole, the difference was not statistically significant. It was concluded that both regimens demonstrated comparable efficacy and similar profiles of adverse effects in treating *H. pylori* infection.

## Introduction

*Helicobacter pylori* (*H. pylori*), a gram-negative bacillus, is widely prevalent worldwide, with an estimated seroprevalence of 50%. Prevalence rates are higher in less developed countries (85-95%), compared to developed countries (30-50%) [1,2]. *H. pylori* infection is widely recognized as the principal etiological factor in the development of peptic ulcer disease, gastritis, and gastric carcinoma. Symptoms frequently associated with dyspepsia, such as upper gastrointestinal tract discomfort, bloating, nausea, and a sensation of fullness shortly after beginning to eat, affect a significant number of people globally. While esophagogastroduodenoscopy (EGD) is commonly performed to exclude the presence of organic disease in these individuals, it is often the case that no ulcers are detected, leading to a diagnosis of functional or non-ulcer dyspepsia. Histological examination reveals that approximately 40.85% of these individuals are infected with *H. pylori*, contributing to chronic gastritis [3]. The prevalence rate of *H. pylori* infection in Pakistan is reported to be 58% in patients with non-ulcer dyspepsia [4].

All patients with evidence of *H. pylori* infection should be offered eradication therapy based on the best locally available resources [5,6]. Various combinations of antibiotics have been scrutinized for their effectiveness in treating *H. pylori*. The efficacy of the primary triple therapy, which comprises a proton pump inhibitor (PPI) and a duo of antibiotics - commonly amoxicillin paired with either clarithromycin or metronidazole - has been on the decline globally. Research spanning from 2000 to 2013 in Japan indicated a substantial resistance rate to these antibiotics: clarithromycin resistance was observed in 31.1% of cases, metronidazole in 60-70%, and levofloxacin in 30-38% [7,8].

The failure to completely remove *H. pylori* is not solely due to the microorganism's resistance to antimicrobial agents; it is also a consequence of suboptimal acid suppression during the treatment regimen (failing to achieve a consistent gastric pH elevation above 5.7), which can compromise the stability and efficacy of antibiotics in the stomach [9,10]. Vonoprazan represents a novel class of PPIs that block the attachment of K+ ions, thus halting the H+/K+ exchange process. This drug maintains a prolonged and robust suppression of gastric acid. Unlike traditional PPIs, vonoprazan is effective against both the active and the more tenacious forms of the proton pump, offering a more potent and enduring diminution of gastric acid secretion. In addition, the rapid onset of action of vonoprazan and its lack of interaction with CYP2C19 genetic variations distinguish it from other PPIs [11,12].

In the past, several regimens for *H. pylori* eradication have been compared and studied, with varying success rates. The escalation in the incidence of this infection, particularly post the COVID-19 pandemic, attributable to the unwarranted administration of antibiotics and the inadequacy of current therapeutic regimens to achieve eradication, underscores the imperative for devising more efficacious treatment modalities. No study has compared the efficacy of lansoprazole plus bismuth quadruple sequential therapy and vonoprazan plus bismuth quadruple sequential therapy in treating *H. pylori* eradication. This clinical trial compared these protocols to elucidate the most efficacious regimen for *H. pylori* eradication in patients with dyspepsia.

## Materials and methods

A non-randomized clinical trial was conducted at the Department of Gastroenterology, Lahore General Hospital, Lahore, Pakistan, following ethical clearance from the institutional review board (AMC/PGMI/LGH/265/01/03/2022). The study spanned 18 months, from March 2022 to August 2023. A cohort of 252 patients who met the inclusion criteria was recruited using a non-probability consecutive sampling method after obtaining informed written consent. The sample size was determined based on a pilot study that predicted an *H. pylori* eradication rate of 95% in the vonoprazan group compared to 84% in the lansoprazole group, with a 95% confidence level and 80% power. A detailed medical history of patients related to age, smoking, residence, previous treatment and NSAID use, and symptoms was documented.

Inclusion criteria

Patients aged 18 to 60 years presenting with dyspepsia, as defined by Rome IV criteria, including symptoms like postprandial fullness, early satiety, epigastric pain, or burning, significant enough to impact daily activities and occurring at least three days weekly for the past three months. Patients underwent esophagogastroduodenoscopy and a urea breath test to confirm *H. pylori* non-ulcer dyspepsia. Eligibility extended to those with mild erythema or gastritis but without endoscopic evidence of ulceration, erosion, or mucosal inflammation in the upper gastrointestinal tract. Patients were required to have a positive urea breath test, conducted two weeks post-cessation of PPIs and four weeks following antibiotic therapy.

Exclusion criteria

Patients were excluded if they had active peptic ulcers with complications, a history of gastrectomy or significant gastrointestinal surgery, allergies or intolerance to study medications, significant comorbidities like diabetes, liver or kidney diseases, were pregnant or breastfeeding, or had a history of alcohol or drug abuse.

Patients were divided into two groups, i.e., Group A and Group B. Group A patients received cap. lansoprazole 30 mg BD + cap. amoxicillin 1 gm BD + tab. tinidazole 500 mg BD + tab. colloidal bismuth subcitrate (2) BD for the first seven days, followed by cap. lansoprazole 30 mg BD + tab. levofloxacin 500 mg BD + tab. azithromycin 500 mg BD + tab. colloidal bismuth subcitrate (2) BD. Group B patients received tab. vonoprazan 20 mg BD + cap. amoxicillin 1 gm BD + tab. tinidazole 500 mg BD + tab. colloidal bismuth subcitrate (2) BD for the first seven days, followed by tab. vonoprazan 20 mg BD + tab. levofloxacin 500 mg BD + tab. azithromycin 500 mg BD + tab. colloidal bismuth subcitrate (2) BD. After initiation of the treatment, all patients were advised to return for follow-ups at seven and 14 days to record any adverse events and assess compliance. Treatment compliance was defined as a patient has taken at least 90% of the prescribed medication. Four weeks post-treatment completion, a repeat urea breath test was conducted to evaluate the efficacy of the treatment.

Data analysis was executed using SPSS version 26.0 IF006 (IBM Corp. Armonk, NY, USA). In the data analysis phase, a pre-protocol analysis was performed on 230 patients, as the final data excluded 22 patients who were lost to follow-up or non-compliant with medication. Numeric variables, such as age and BMI, were presented as means with standard deviation (SD), while frequency and percentages were utilized for categorical variables like gender, BMI category, history of previous medications and NSAID use, smoking status, adverse events, and efficacy. The efficacy outcomes were compared using the chi-square test or Fisher's exact test, considering a p-value of ≤0.05 indicative of statistical significance.

## Results

The study initially recruited 252 individuals; however, 22 participants were subsequently removed from the analysis. In Group A (lansoprazole), six patients were lost to follow-up, and five exhibited non-compliance. In Group B (vonoprazan), eight did not attend the follow-up, and three were non-compliant, resulting in 115 participants per group for the final analysis. In the study, the participants' mean age and BMI were 43.71± 14.10 years and 27±4.05 Kg/m^2^, respectively. The lansoprazole treatment group had a mean age of 44.61±13.20 years and a BMI of 27.38±4.12 kg/m², while the vonoprazan group had a mean age of 42.81±14.95 years and a BMI of 26.60±3.97 kg/m², indicating no significant differences in age (p = 0.334) or BMI (p = 0.148) between the two groups. The study observed no significant disparities in baseline demographics, lifestyle, and clinical characteristics between Group A (lansoprazole) and Group B (vonoprazan), with p-values ranging from 0.126 to 0.866 (Table 1). 

**Table 1:** Comparison of baseline characteristics among the treatment groups BMI: body mass index, NSAID: nonsteroidal anti-inflammatory drug, PPI: proton pump inhibitor

Baseline characteristics	Category	Lansoprazole Group (Group A) n = 115	Vonoprazan Group (Group B) n = 115	Chi-square (p-value)
Age	<40 years	40 (34.8%)	51 (44.3%)	0.138
	≥40 years	75 (65.2%)	64 (55.7%)	
Gender	Male	63 (54.8%)	56 (48.7%)	0.356
	Female	52 (45.2%)	59 (51.3%)	
BMI	<24.9	41 (35.7%)	43 (37.4%)	0.126
	25.0-29.9	35 (30.4%)	-	
	≥30.0	39 (33.9%)	26 (22.6%)	
Residence	Rural	45 (39.1%)	40 (34.8%)	0.495
	Urban	70 (60.9%)	75 (65.2%)	
Drinking water source	Tap	67 (58.3%)	72 (62.6%)	0.500
	Filtered	48 (41.7%)	43 (37.4%)	
Meal source habit	Homemade	93 (80.9%)	94 (81.7%)	0.866
	Restaurant	22 (19.1%)	21 (18.3%)	
Smoking		21 (18.3%)	20 (17.4%)	0.863
History of NSAID use		15 (13.0%)	14 (12.2%)	
Paan/gutka use		13 (11.3%)	20 (17.4%)	0.188
History of PPI use		57 (49.6%)	68 (59.1%)	0.145
History of antibiotic use		31 (27.0%)	23 (20.0%)	0.213
Previous treatment history	Complete	21 (18.3%)	16 (13.9%)	0.456
	Partial	10 (8.7%)	7 (6.1%)	
	None	84 (73.0%)	92 (80.0%)	

**Figure 1:**
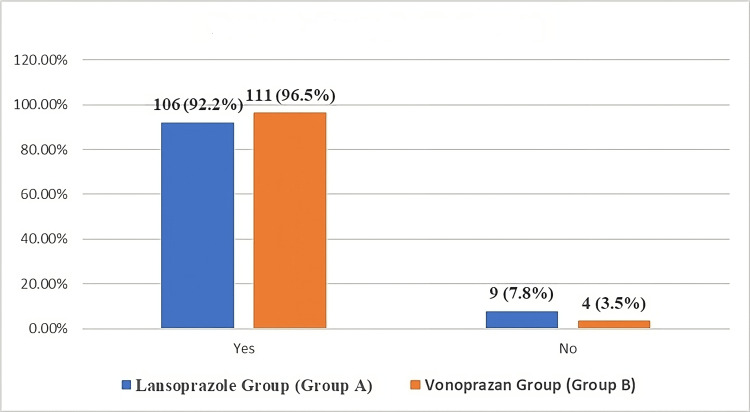
Comparison of efficacy (eradication rate) among the Helicobacter pylori eradication treatment groups.

In comparing treatment efficacy, Group A achieved an efficacy of 92.2%, whereas Group B had a slightly higher success rate of 96.5%. However, this difference was insignificant (p-value = 0.153) (Figure 1). 

*H. pylori* eradication was uniformly high among the patients, indicating no notable differences in the treatment efficacy across the subgroups (Table 2).

**Table 2:** Comparative analysis of Helicobacter pylori eradication efficacy across baseline patient characteristics. BMI: body mass index, NSAID: nonsteroidal anti-inflammatory drug, PPI: proton pump inhibitor

Baseline characteristics	Category	Efficacy (Yes) n = 217	Efficacy (No) n = 13	p-value
Age group (years)	<40	89 (98.9%)	2 (1.1%)	0.066
	≥40	128 (92.1%)	11 (7.9%)	
Gender	Male	110 (92.4%)	9 (7.6%)	0.194
	Female	107 (96.4%)	4 (3.6%)	
Smoking		39 (95.1%)	2 (4.9%)	0.813
Paan/gutka use		30 (90.9%)	3 (9.1%)	0.355
Residence	Rural	78 (91.8%)	7 (8.2%)	0.194
	Urban	139 (95.9%)	6 (4.1%)	
History of NSAID	Yes	27 (93.1%)	2 (6.9%)	0.756
Drinking water source	Tap	129 (92.8%)	10 (7.2%)	0.211
	Filtered	88 (96.7%)	3 (3.3%)	
Meal source habit	Homemade	177 (94.7%)	10 (5.3%)	0.677
	Restaurant	40 (93.0%)	3 (7.0%)	
BMI (Kg/m^2^)	<24.9	81 (96.4%)	3 (3.6%)	0.108
	25.0-29.9	78 (96.3%)	3 (3.7%)	
	≥30.0	58 (89.2%)	7 (10.8%)	
History of antibiotic use		51 (94.4%)	3 (5.6%)	0.972
History of PPI		117 (93.6%)	8 (6.4%)	0.592
Previous treatment history	Complete treatment	36 (94.7%)	2 (5.3%)	0.408
	Partial treatment	15 (88.2%)	2 (11.8%)	
	No treatment	166 (94.3%)	10 (5.7%)	

Table 3 delineates the adverse effects reported by patients in both treatment cohorts. 

**Table 3:** Comparison of adverse effects among the treatment groups.

Adverse effects	Lansoprazole group (Group A) n = 115	Vonoprazan group (Group B) n = 115	Chi-square (p-value)
Headache	8 (7.0%)	5 (4.3%)	0.392
Nausea	8 (7.0%)	6 (5.2%)	0.581
Diarrhea	5 (4.3%)	8 (7.0%)	0.392
Constipation	6 (5.2%)	8 (7.0%)	0.581
Abdominal pain	9 (7.8%)	15 (13.0%)	0.196
Flatulence	7 (6.1%)	6 (5.2%)	0.775
Overall adverse effects	17 (14.8%)	19 (16.5%)	0.717

## Discussion

In the comparative analysis of quadruple sequential therapy for dyspepsia due to *H. pylori*, the therapeutic efficacy of vonoprazan and lansoprazole was similar. This study demonstrated that the eradication rates were high for both groups, with Group A (lansoprazole) showing a 92.2% success rate and Group B (vonoprazan) having a marginally higher rate at 96.5% (p = 0.153). The study's strength is further supported by the homogeneity of baseline characteristics between the two groups, ensuring that variables, such as age, BMI, and gender, and lifestyle factors, such as smoking status and meal habits, did not confound the results. The adherence to treatment and follow-up was reasonably well maintained, with only a minority of participants being lost to follow-up or non-compliant, showing that both treatment regimens were well tolerated. This study's treatment regimes were designed after an extensive literature review. This evaluation included the comparison of the efficacy of different PPIs and vonoprazan, emerging resistance to first-line clarithromycin-containing regimes, the potential role of quadriple bismuth-containing regimes, varying treatment durations and patterns ranging from the standard courses to more sequential therapies in eradicating *H. pylori* infection.

An extensive systematic review and meta-analysis, encompassing 178 studies, indicates a global prevalence of significant *H. pylori* antibiotic resistance. Initial and subsequent resistance levels to clarithromycin, metronidazole, and levofloxacin are substantial (15% or greater) across the majority of regions. Specifically, resistance to clarithromycin is prevalent in European, Eastern Mediterranean, and Western Pacific regions, with comparatively lower rates observed in the Americas and South-East Asia. Notably, the presence of clarithromycin resistance has been linked to unsuccessful *H. pylori* eradication efforts [6]. A meta-analysis of 46 randomized trials compared sequential therapy to alternate regimens. *H. pylori's* eradication rate was high for sequential therapy (84%) compared to alternate regimes [13].

In a retrospective study, Howden et al. (2023) observed lower *H. pylori* treatment rates and costs with vonoprazan compared to PPIs [14]. Shinozaki et al. (2021) analyzed 16 studies, suggesting vonoprazan superiority in the eradication of *H. pylori* compared to PPIs (OR 1.51, 95% CI 1.27-1.81, p < 0.001) with no significant adverse event differences [15]. Zuberi et al. (2022) reported higher eradication with vonoprazan dual therapy (93.5%) versus omeprazole triple therapy (83.9%, p = 0.042) [16]. Yang et al. (2023) showed eradication rates of 96.2% for 10-day, 94.9% for 14-day vonoprazan, and 93.6% for esomeprazole treatments [17]. Shichijo et al. (2016) observed higher first-line eradication with vonoprazan (87.2%) compared to PPIs (72.4%, p < 0.01) [18].

While analyzing the literature related to the comparison of azithromycin therapies with other alternate regimes, it was found that azithromycin-based regimens demonstrate comparable efficacy and lower adverse effects in *H. pylori* eradication. A multicenter randomized controlled trial (RCT) study from Russia conducted by Ivashkin et al. (2002) found that omeprazole, azithromycin, and amoxicillin (OAA) achieved a 76% eradication rate, significantly higher than omeprazole, amoxicillin, and metronidazole (OAM) at 26% (p < 0.001) [19]. Similarly, Sullivan et al. (2002) resulted in an 84.6% eradication rate with bismuth, lansoprazole, azithromycin, and clarithromycin (B-LAC) compared to 52% with bismuth, lansoprazole, and azithromycin (B-LAA) [20]. A meta-analysis reported that adverse effects were comparably low in the azithromycin group compared to the non-azithromycin group at 15.8% and 25%, respectively [21]. Mohiuddin et al. (2021) found a slightly higher eradication rate and better compliance in the azithromycin group than in clarithromycin [22]. Contrarily, Sarkeshikian et al. (2013) reported an 83% efficacy with clarithromycin-based triple therapy versus 75% with azithromycin in functional dyspepsia (p = 0.158) [23].

In studies evaluating the role of bismuth in *H. pylori* treatment, Huh et al. (2022) found that both lansoprazole and vonoprazan-based bismuth quadruple therapies achieved 100% eradication [24]. Similarly, a meta-analysis revealed that bismuth-containing quadruple therapies were superior to conventional triple therapy [25]. Meanwhile, an RCT by Ebrahimi et al. reported no significant difference in eradication rates between regimes with or without bismuth (64.15% vs. 79.2%, p = 0.13) [26]. Several studies have documented that incorporating bismuth compounds into eradication regimens significantly boosts the response rate among infected patients by reducing the density of bacteria [27,28]. 

The study acknowledges certain limitations. Primarily, the non-randomized nature and employment of a consecutive sampling technique could potentially allow for the introduction of selection bias. However, a thorough subgroup analysis within both treatment arms has disclosed no substantial differences, suggesting that any potential selection bias did not significantly affect the comparative outcomes of the study groups. Although the study was conducted at a tertiary care center known for its advanced gastroenterology department and receiving referrals from all over Punjab with ample sample size and study duration, the results may not be entirely applicable to the general population. This limitation demands that there is a need for a multicenter study to verify and broaden the applicability of our results across diverse populations and healthcare settings. Lastly, the practicality of these findings might be restricted in regions where bismuth preparation is not readily available. For future research, adopting an RCT design would be beneficial to minimize potential biases further and enhance the robustness of the findings. In addition, exploring patient subgroups based on predispositions or variations in their microbiome could provide valuable insights into creating personalized treatment approaches. It is also recommended that long-term follow-up studies be conducted to evaluate the recurrence of infection and the sustainability of treatment efficacy. Such endeavors will validate our findings and expand the understanding of *H. pylori* eradication therapies, ultimately contributing to more effective and tailored treatment strategies.

## Conclusions

In a comparative study of vonoprazan and lansoprazole-based quadruple sequential therapy, conducted with 252 patients having non-ulcer dyspepsia due to *H. pylori* infection, both treatment groups demonstrated high eradication rates. Group A (lansoprazole) achieved a 92.2% success rate, while Group B (vonoprazan) reached 96.5%. Despite these high rates, the difference between the two treatments was not statistically significant (p = 0.153). The adverse effects profile was comparable between the two groups, showing insignificant differences (p = 0.717). The study, however, faced limitations, such as lost to follow-up and non-compliance. Nonetheless, the results suggest both treatments as effective options for managing this condition, providing valuable insights for clinicians in choosing appropriate therapeutic strategies.
